# Human Milk Oligosaccharide 2′-Fucosyllactose Improves Innate and Adaptive Immunity in an Influenza-Specific Murine Vaccination Model

**DOI:** 10.3389/fimmu.2018.00452

**Published:** 2018-03-09

**Authors:** Ling Xiao, Thea Leusink-Muis, Nienke Kettelarij, Ingrid van Ark, Bernadet Blijenberg, Nienke A. Hesen, Bernd Stahl, Saskia A. Overbeek, Johan Garssen, Gert Folkerts, Belinda van’t Land

**Affiliations:** ^1^Utrecht Institute for Pharmaceutical Sciences (UIPS), Utrecht University, Utrecht, Netherlands; ^2^Department of Immunology, Human Milk Research, Nutricia Research, Utrecht, Netherlands; ^3^Department of Paediatric Immunology, Wilhelmina Children’s Hospital, University Medical Center Utrecht, Utrecht, Netherlands

**Keywords:** vaccination, diet, 2′fucosyllactose, immune response, B-cell activation, delayed-type hypersensitivity, human milk oligosaccharides

## Abstract

**Background:**

Human milk is uniquely suited to provide optimal nutrition and immune protection to infants. Human milk oligosaccharides are structural complex and diverse consisting of short chain and long chain oligosaccharides typically present in a 9:1 ratio. 2′-Fucosyllactose (2′FL) is one of the most prominent short chain oligosaccharides and is associated with anti-infective capacity of human milk.

**Aim:**

To determine the effect of 2′FL on vaccination responsiveness (both innate and adaptive) in a murine influenza vaccination model and elucidate mechanisms involved.

**Methods:**

A dose range of 0.25–5% (w/w) dietary 2′FL was provided to 6-week-old female C57Bl/6JOlaHsd mice 2 weeks prior primary and booster vaccination until the end of the experiment. Intradermal (i.d.) challenge was performed to measure the vaccine-specific delayed-type hypersensitivity (DTH). Antigen-specific antibody levels in serum as well as immune cell populations within several organs were evaluated using ELISA and flow cytometry, respectively. In an *ex vivo* restimulation assay, spleen cells were cocultured with influenza-loaded bone marrow-derived dendritic cells (BMDCs) to study the effects of 2′FL on vaccine-specific CD4+ and CD8+ T-cell proliferation and cytokine secretions. Furthermore, the direct immune regulatory effects of 2′FL were confirmed using *in vitro* BMDCs T-cell cocultures.

**Results:**

Dietary 2′FL significantly (*p* < 0.05) enhanced vaccine specific DTH responses accompanied by increased serum levels of vaccine-specific immunoglobulin (Ig) G1 and IgG2a in a dose-dependent manner. Consistently, increased activation marker (CD27) expression on splenic B-cells was detected in mice receiving 2′FL as compared to control mice. Moreover, proliferation of vaccine-specific CD4+ and CD8+ T-cells, as well as interferon-γ production after *ex vivo* restimulation were significantly increased in spleen cells of mice receiving 2′FL as compared to control mice, which were in line with changes detected within dendritic cell populations. Finally, we confirmed a direct effect of 2′FL on the maturation status and antigen presenting capacity of BMDCs.

**Conclusion:**

Dietary intervention with 2′FL improves both humoral and cellular immune responses to vaccination in mice, which might be attributed in part to the direct effects of 2′FL on immune cell differentiation.

## Introduction

Vulnerable populations with an immature or incompetent immune system (such as infants, preterm born infants, immune compromised individuals, and elderly) renders them at increased risk for infections while limited responsiveness to vaccines is often detected (1). For example, early-life vaccination remains a challenge due to a range of limitations in generating appropriate adaptive and innate immune responses. Neonates have weak and short-lived antibody responses [reviewed in Ref. (1)] which is insufficient to totally prevent viral replication and infection. In addition, the neonatal immunological milieu is polarized toward a Th2-type immunity with dampening of Th1-type immune responses [e.g., interferon-γ (IFN-γ) production] and cytotoxicity (CD8+ T-cell) (2, 3), resulting in qualitatively and quantitatively limited cellular responses to infections compared to older infants. Strategies aimed at inducing protective humoral and optimal cellular immune responses may thus facilitate the improvement of neonatal vaccine efficacy. Moreover, recent evidence indicate that also the modification of the gut microbiota composition influences influenza vaccine responsiveness, which provide new insights on the regulation of the immune responses following (influenza) vaccination (4). Taken together, dietary interventions that can improve the immune system and shape an optimal gut microbiota is a promising strategy to improve vaccine efficacy.

Diet is one of the major environmental factors that affect both immune development and gut microbiota composition and function (5, 6). The beneficial properties of human milk against immune-related disorders and infectious diseases have been widely reported and can be attributed to bioactive components of human milk such as human milk oligosaccharides (HMOS). HMOS are the third most abundant components in human milk and constitute to the defense system of human milk due to their well-known prebiotic effects and potent properties to directly interact with the immune cells (7). HMOS are comprised of a mixture existing over 200 specific oligosaccharides, consisting of short chain and long chain structures typically in a ratio of 9:1, respectively. The most predominant oligosaccharide (representing up to 20% of total HMOS) in the milk of fucosyltransferase 2 (FUT2) gene positive mothers (termed “secretors”) is 2′-fucosyllactose (2′FL) (7).

2′FL is synthesized through enzymatic transfer of fucose guanosine 5′-diphosphate-l-fucose to lactose by FUT2, and is suggested to contribute to the health benefits of the complex HMOS mixture (8). Specifically, anti-adherent effects on pathogens (9) as well as prebiotic properties on mutualist microbes have been described for some HMOS structures (10). In addition, the complex HMOS mixture indirectly leads to the regulation of the mucosal immune system *via* different mechanisms such as short chain fatty acids (SCFAs)-mediated activity. Interestingly, recent evidence showed that specific HMOS, including 2′FL is detected in the systemic circulation after oral administration (11), suggesting potential biological functions for HMOS beyond serving as preferential bacterial substrates devoted to maintaining an optimal gut microbiota. Within the mucosa, dendritic cells (DCs) are located just below the epithelial layer. DCs are major determinants of immunity due to their role in the initiation of long-term adaptive immunity by detecting and presenting antigens to CD4+/8+ T-cells (12, 13) and are novel targets for enhancing vaccination efficacy (14). Using glycan microarrays, 2′FL has been demonstrated to bind to DC-specific intercellular adhesion molecule-3-grabbing non-integrin (DC-SIGN) (15), suggesting that specific HMOS such as 2′FL may directly interact with the innate immune cells subsequently influencing adaptive immunity within infants. Although little evidence can be found for the direct effects of authentic HMOS on the phenotype and/or functions of DCs, it has been demonstrated that a mixture of short-chain galacto-oligosaccharides (scGOS) and long-chain fructo-oligosaccharides (lcFOS) at a ratio of 9:1, which was designed to resemble the molecular size distribution and functional aspects of the neutral fraction of authentic HMOS, directly influences the development of human monocytes derived DCs as observed by their phenotypes, cytokine production, and T-cell priming capacity *in vitro* (16). *In vivo*, specific prebiotic oligosaccharides effectively improved vaccine-specific immune responses in a Th1-dependent fashion using several murine vaccination models (17–20). Therefore, we hypothesized that 2′FL, which is structurally identical to a specific authentic HMO, could induce direct immunomodulatory effects in a murine vaccination model and thereby improving the vaccine responsiveness.

## Materials and Methods

### Mice

Six-week-old female C57Bl/6JOlaHsd mice were purchased from Envigo (Horst, The Netherlands) and housed in the animal facility of Utrecht University and kept under normal conditions, with a 12 h dark and light cycle with access to food and water *ad libitum*. The animals received standard diets and routine care for a week upon arrival in the animal facility, before the start of the experiments. All experiments were approved by the Animal Ethics Committee of Utrecht University (approval number DEC 2015.II.243.038).

### Vaccination Protocol

**Figure 1:**
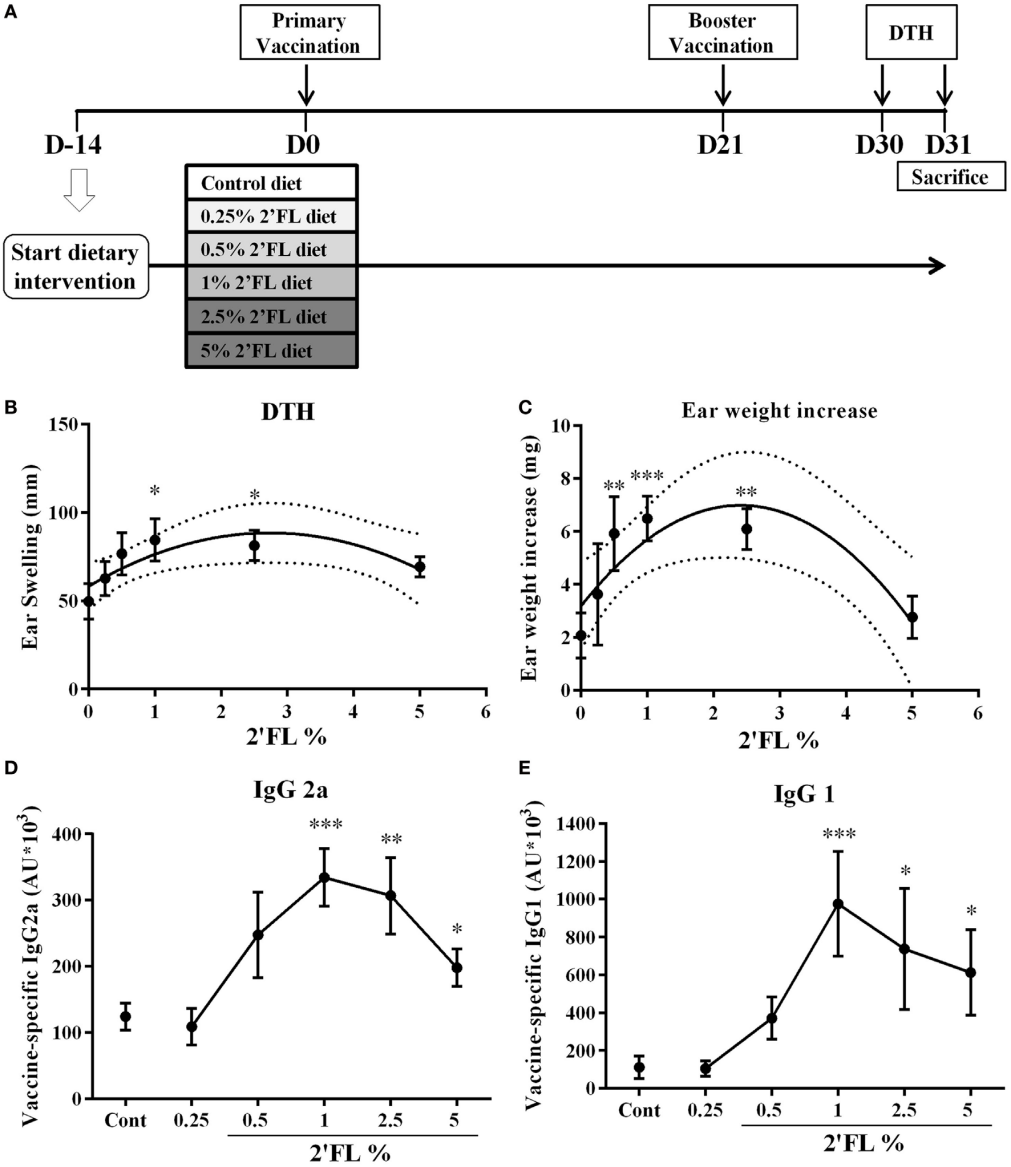
Effect of dietary intervention with 2′-fucosyllactose (2′FL) on vaccine-specific delayed-type hypersensitivity (DTH) responses and antibody levels in serum collected at day 31. **(A)** Schematic overview of the study design (SDI = Start Dietary Intervention). **(B)** DTH response and **(C)** ear weight difference between left (ear challenge with PBS) and right (ear challenge with influvac) ear at 24 h after ear challenge. Vaccine-specific **(D)** IgG2a and **(E)** IgG1 levels in serum measured by means of ELISA assays. Data are presented as mean ± SEM *n* = 7–9/group in **(B–E)**. In panels **(B,C)**, quadratic regression was performed on Graph pad 7 to analyzes dose-response relationship and the optimal fitting curve is plotted in the graph together with the 95% CI’s. In panels **(D,E)** one-way ANOVA followed by Bonferroni’s *post hoc* test was used for selected groups. *p < 0.05, **p < 0.01, ***p < 0.001, indicate statistical differences detected compared to vaccinated control group.

As depicted in Figure 1A, after 1 week acclimation period, mice were fed the control (AIN93G) or the 2′FL containing diet until the end of the experiment (*n* = 9 per group). Concentrations of oligosaccharides in human milk show variations that are dependent on both the secretor type of the mother and the lactation period. Human milk may contain up to 20 g/L total oligosaccharides, of which on average 2.7 g/L 2′FL is detected, depending on detection method (7). Semisynthetic AIN93G diets were mixed with 0.25, 0.5, 1, 2.5, or 5% 2′FL (SSNIFF Spezialdiäten GmbH, Germany) in order to allow testing the effects of 2′FL starting from a human physiological concentration (produced by microbial fermentation, with >90% purity) on vaccine-induced immune responses. The percentages of 2′FL were exchanged against equal amount (wt/wt) total carbohydrates present in the control diet. Subcutaneous (s.c.) vaccination was conducted using Influvac (Abbott Biologicals B.V., Weesp, The Netherlands) from season 2015/2016. This inactivated influenza virus vaccine is based on isolated hemagglutinin (HA) and neuraminidase antigens of three strains of myxovirus influenza, in a dose equivalent to 30 µg/mL HA per strain (90 µg/mL HA in total). Under isoflurane anesthesia, the mice received a primary vaccination and a booster vaccination in a total volume of 100 µL, the vaccine concentration (90 µg/mL). Concentration used was based on our pilot experiment for vaccine titration (data not shown). The booster vaccination was given 21 days after the primary vaccination. The experiments ended 10 days after booster vaccination. Negative control group (*n* = 3) that was included in all experiments (indicated with “sham group”) received injections with 100 µL PBS. Sham group was not used for statistical comparisons to supplemented groups but serves to demonstrate the specificity of vaccine-induced responses. Delayed-type hypersensitivity (DTH) reactions were induced 9 days after booster vaccination, by i.d. injection of 20 µL Influvac into the ear pinnae of the right ear, and 20 µL PBS into the ear pinnae of left ears as basal line. Ear thickness was measured in duplicate before vaccine challenge and 24 h thereafter, using a digital-micrometer (Mitutoyo Digimatic 293561, Veenendaal, The Netherlands). The antigen specific DTH responses were calculated by the formula: DTH = Right ear (Thickness @24 h − @0 h) − Left ear (Thickness @24 h − @0 h). In addition, after sacrificing the animals, both ears were punched with a sharped metal borer for the measurement of Ear weight. Ear weight increase = Right ear (Weight) − Left ear (Weight).

### Vaccine-Specific Immunoglobulins (Igs) in Serum

Blood was collected *via* cheek puncture at the end of the experiment prior to sectioning of the mice. Blood samples were centrifuged (10,000 rpm for 10 min) and serum was stored at −20°C until analysis of vaccine-specific antibodies by means of ELISA. Determination of vaccine-specific antibodies was performed as described previously (17). Briefly, 96-well plates (Costar EIA/RIA plate, Alphen a/d Rijn, The Netherlands) were coated with 1:100 diluted Influvac in PBS. Blocking reagent was 2% BSA (Sigma, Zwijndrecht, The Netherlands) in PBS. A dilution series of pooled serum that contained vaccine specific antibodies was added for standard curve calculation. Per mouse 10 µL serum was used to determine antibody levels with a final dilution of 14,700× by a serial dilution. Anti-IgG1-biotin and anti-IgG2a-biotin (Becton Dickinson, Heerhugowaard, The Netherlands) antibodies were diluted 1:1,000 in dilution buffer. For the biotin-conjugated antibodies, the plates were subsequently incubated with a 1:20,000 dilution of streptavidin-HRP (Biosource, Etten-Leur, The Netherlands). Optical density was measured with a Benchmark microplate reader (BioRad, Hercules, CA, USA) at a wavelength of 490 nm. Concentrations in test sera were calculated in arbitrary units (AU), relative to the standard curve of the diluted pooled serum. The top concentration of the pooled serum was defined as 100 AU/mL.

### Flow Cytometry of Immune Cells

Freshly isolated spleen and mesenteric lymph node (MLN) cells were obtained and resuspended in PBS/1% bovine serum albumin and incubated with anti-mouse CD16/CD32 (Mouse BD Fc Block; BD Pharmingen, San Jose, CA, USA) to block non-specific binding sites for 20 min on ice. For surface staining, cells were incubated with CD4-PerCp-Cy5.5, CD69-PE, CD25-AlexaFluor488, CD11c-PerCp-Cy5.5, CD103-APC, CD40-FITC, CD86-PE-cy7, MHCII-APC, CD3-Percy5.5, CD27-PE, CD19-APC, B220-FITC, NK1.1-APC, or CD49b-FITC (eBiosciences, San Diego, CA, USA). Viable cells were distinguished by means of a fixable viability dye eFluor^®^ 780 (eBioscience), per sample 0.5 × 10^6^ and 1.0 × 10^6^ cells were used for surface and intracellular staining, respectively. More than 80% live cells were obtained in all the stainings. For detecting transcription factors, cells were first fixed and permeabilized with forkhead box protein 3 (Foxp3) Staining Buffer Set (eBioscience) according to manufacturer’s protocol and then stained with Foxp3-PE-cy7, and Tbet-APC (eBioscience). Isotype controls were used for each antibody and fluorescence-minus-one’s controls were used to identify the positive population from the negative population (all Isotypes purchased from eBioscience). In addition, anti-mouse CD16/32 antibody was used as an Fc-block prior to staining the samples (eBioscience). Results were collected with BD FACSCanto II flow cytometer (Becton Dickinson, Franklin Lakes, NJ, USA) and analyzed with FlowLogic software (Inivai Technologies, Mentone, Victoria, Australia).

### Generation of Bone Marrow-Derived Dendritic Cells (BMDCs)

Surplus mice (female, 11-week-old C57BL/6JOlaHsd) were kept in our animal facility at the Central Animal Laboratory (GDL), Utrecht University, The Netherlands. Bone marrow cells were isolated from femurs and cultured in RPMI 1640 medium (Gibco) supplemented with 10% FBS and 100 U/mL penicillin/streptomycin, 10 mM HEPES, 1 mM sodium pyruvate, and Eagles minimum essential medium (MEM) non-essential amino acids (all from Gibco Life Technologies) in the presence of 10 ng/mL GM-CSF (Prosepec, The Netherlands) for 6 days to obtain immature BMDC (iDC). Induced iDCs were then loaded with the vaccine at a concentration of 0.9 µg/mL and incubated for 24 h at 37°C, 5% CO_2_ before coculturing with splenocytes isolated from the vaccinated mice receiving the dietary interventions. iDCs treated with medium were used as negative control.

### *Ex Vivo* Restimulation of Whole Splenocytes with Vaccine-Loaded BMDCs

Fresh spleens were removed aseptically and single cell suspensions were obtained as described above. Splenocytes (5 × 10^6^ cells/mL) were cocultured with DCs (5 × 10^5^ cells/mL) loaded with or without influenza virus in 96-well U-bottom culture plates for 5 days at 37°C, 5% CO_2_, RPMI 1640 medium (Gibco) supplemented with 10% FBS and 100 U/mL penicillin/streptomycin, 10 mM HEPES, 1 mM sodium pyruvate and Eagles MEM non-essential amino acids was used (all from Gibco Life Technologies). Supernatants were collected at day 5 and stored at −80°C until use and analyzed for the concentration of IL-10, IL-17A, IL-13, IL-2, and IL-4 by Bead-based immunoassays (Boiolgend LGENDplex, UK, London) and IFN-γ (eBioscience, The Netherlands) and TNF-α (eBioscience, The Netherlands) by ELISA assays according to manufacturer’s instructions.

### CFSE Labeling and Coculture of Whole Splenocytes with Vaccine-Loaded BMDCs

In parallel, splenocytes were labeled with cell trace dye CFSE (Thermo Fisher, The Netherlands) according to the manufacturers’ instructions at a final concentration of 1 µM and FITC-positive cells were acquired, and were cocultured with DCs loaded with or without influenza virus at 10:1 in 96-well U-bottom culture plates for 5 days at 37°C, 5% CO_2_. On day 5, the mix of cocultured cells was stained with viability dye, CD4-APC and CD8-PE (eBioscience, The Netherlands). Proliferation of splenocytes was determined by setting gates on proliferated cells and comparing the percentage of proliferated cells from 2′FL mice with vaccinated control mice.

### qPCR Analysis

Ileum samples from influenza-vaccinated mice sacrificed after DTH measurement were collected in RNAlater (Invitrogen, Thermo Fisher Scientific, Waltham, MA, USA) and over night stored in the fridge, after which they were stored at −80°C until mRNA isolation. Before mRNA isolation, tissues were homogenized by using Precellys^®^ CKMix Tissue Homogenizing Kit (Bertin, KT039611009.2) with the Precellys 24 homogenizer (Bertin, EQ 03119-200-RD000.0). mRNA was extracted using a NucleoSpin^®^ RNA Plus kit (Macherey-Nagel, 740984.250) in combination with the rDNAse set (Macherey-Nagel, #740963) to remove contaminating DNA. cDNA was synthesized using an iScript™ advanced kit (Bio-Rad, Basel, Switzerland) according to the manufacturer’s protocol in the PTC-100 Programmable Thermal Controller (MJ research, PTC-100). cDNA and mRNA samples were stored at −80°C. Quantitative analysis was performed on a CFX96 Real-Time C1000 Thermal Cycler detection system with the use of an IQ™ SYBR^®^ Green Supermix according to manufacturer’s protocol (both from Bio-Rad). Custom designed primers were made for CD40, CD80, CD86, PD-L1, TGF-β3, TNF-α, IL-1α, IL-1β, IL-10, IL-12β, CXCL9, CXCL10, FUT2, FUT8, and reference gene GAPDH (Biolegio, Nijmegen, The Netherlands, gene sequences and annealing temperatures are listed in Table 1). 300 nM forward and reverse primers were used in 10 µL reactions (8.8 µL mix + 1.2 µL cDNA), which were measured in a 96 well Hard-Shell PCR plate with thin walls (Bio-Rad, HSP9601). The plate was covered with a Microseal “B” film (Bio-Rad, MSB1001) and then measured at the appropriate temperature for 40 cycles in the CFX96 Real-Time System C1000 Thermal Cycler (Bio-Rad, 1855195). All samples were measured in triplicate, values within each triplicate that differed ≥0.5 CT were removed. The average of the remaining values was taken for each mouse. Relative mRNA expression of each mouse was calculated with the Livak method (2−ΔΔCT) (21) and depicted as a fold change of the average of control group (onefold).

**Table 1:** Sequences of specific primers for detected genes with corresponding accession number.

Gene ID	Accession number	Forward primer sequence (5′–3′)	Reverse primer sequence (5′–3′)
FUT2	NM_018876.4	CCCACTTCCTCATCTTTGTCTTT	TTTGAACCGCCTGTAATTCCTT
FUT8	NM_016893.5	CAGGGGATTGGCGTGAAAAAG	CGTGATGGAGTTGACAACCATAG
IL-10	NM_010548.2	TGAATTCCCTGGGTGAGAAGC	CACCTTGGTCTTGGAGCTTATT
TGF-β3	NM_009368.3	GCTGTTGAGGAGAGAGTCCA	TCCATTGGGCTGAAAGGTGT
IL-12β	NM_001303244.1	GTAACCAGAAAGGTGCGTTCC	GAACACATGCCCACTTGCTG
IL-1α	NM_010554.4	ATGAAGCTCGTCAGGCAGAAG	GAGATAGTGTTTGTCCACATCCTGAT
IL-1β	NM_008084.2	ATCCCAAGCAATACCCAAAGAA	GCTGATGTACCAGTTGGGGAA
TNF-α	NM_013693.3	AACGGCATGGATCTCAAAGA	TTTCTCCTGGTATGAGATAGCAAATC
CXCL9	NM_008599	TCTGCCATGAAGTCCGCTG	CAGGAGCATCGTGCATTCCT
CXCL10	NM_021274.2	GCCGTCATTTTCTGCCTCAT	GCTTCCCTATGGCCCTCATT
CD80	NM_009855.2	TGGGAAAAACCCCCAGAAGAC	GCCCGAAGGTAAGGCTGTT
CD86	NM_019388.3	CAGCACGGACTTGAACAACC	CTCCACGGAAACAGCATCTGA
CD40	NM_011611.2	ACTAATGTCATCTGTGGTTTAAAGT	GAAACACCCCGAAAATGGT
PD-L1	NM_021893.3	TGATCATCCCAGAACTGCCTG	AGGAGGACCGTGGACACTAC

### Treatment of BMDCs

Immature BMDCs were generated as described in Section “Generation of Bone Marrow Derived Dendritic Cells.” 2′FL (1.25–10 mg/mL) was added to the iDCs and incubated for 24 h at 37°C, 5% CO_2_. iDCs treated with medium were used as negative control and LPS (100 ng/mL) was added to mature the iDCs and serves as a positive control. Phenotypes of DCs were identified using flow cytometry measuring surface marker MHC-I, MHC-11, CD40, and CD86. In addition, IL-10 and IL-12p70 levels in the supernatant were assessed using ELISA kits (eBioscience, The Netherlands) according to manufacturer’s protocol.

### Statistical Analysis

All data were analyzed using GraphPad Prism 7.0 software for Macintosh (GraphPad Software, San Diego, CA, USA). Dose–response relationships analysis was described in our previous study (17). All other data sets were checked for normality in distribution (and normalized using log transformation) after which the data were analyzed by one-way ANOVA, followed by a Bonferroni’s multiple comparison *post hoc* test for selected comparisons. Data are presented as mean ± SEM. *p < 0.05, **p < 0.01, ***p < 0.001, and ****p < 0.0001 were considered statistical significant.

## Results

### Dietary 2′FL Improved Vaccine-Specific Cellular and Humoral Response

In C57BL/6 mice (*n* = 9 per group) receiving twice s.c. influvac vaccination, a dietary range between 0.25 and 5% of 2′FL (w/w of total diet) was provided and antigen specific vaccination responses were analyzed (Figure 1A). A dose-dependent increase in the influenza-specific DTH response (measured as ear swelling and ear weight gain) was detected in mice receiving 2′FL as compared to vaccinated mice receiving control diet (Figures 1B,C). Quadratic regression was performed and identified a linear increase in DTH up to 1% providing a pharmaceutical optimum at 2.5% 2′FL. More specifically, dietary intervention with 1% 2′FL significantly increased DTH response (*p* < 0.05) and ear weight gain (*p* < 0.001) in the vaccinated mice receiving 2′FL compared to control diet. Vaccine-specific humoral immune responses were detected in serum obtained at the end of the experiment (day 31). Subclass specific IgG2a and IgG1-antibody concentrations were detected in serum of vaccinated mice receiving control diet and compared to sham-vaccinated mice indicating that induced antibodies are vaccine-specific (data not shown). Furthermore, in serum of vaccinated mice receiving dietary 2′FL increased levels of both vaccine-specific IgG2a (*p* < 0.001 for 1% 2′FL, *p* < 0.01 for 2.5% 2′FL, and *p* < 0.05 for 5% 2′FL) and IgG1 (*p* = 0.0945 for 0.5% 2′FL, *p* < 0.001 for 1% 2′FL, *p* < 0.05 for 2.5% 2′FL, *p* < 0.05 for 5% 2′FL) were detected as compared to mice receiving control diet (Figures 1D,E).

### Dietary 2′FL Increased B-Cell Activation and Frequency

**Figure 2:**
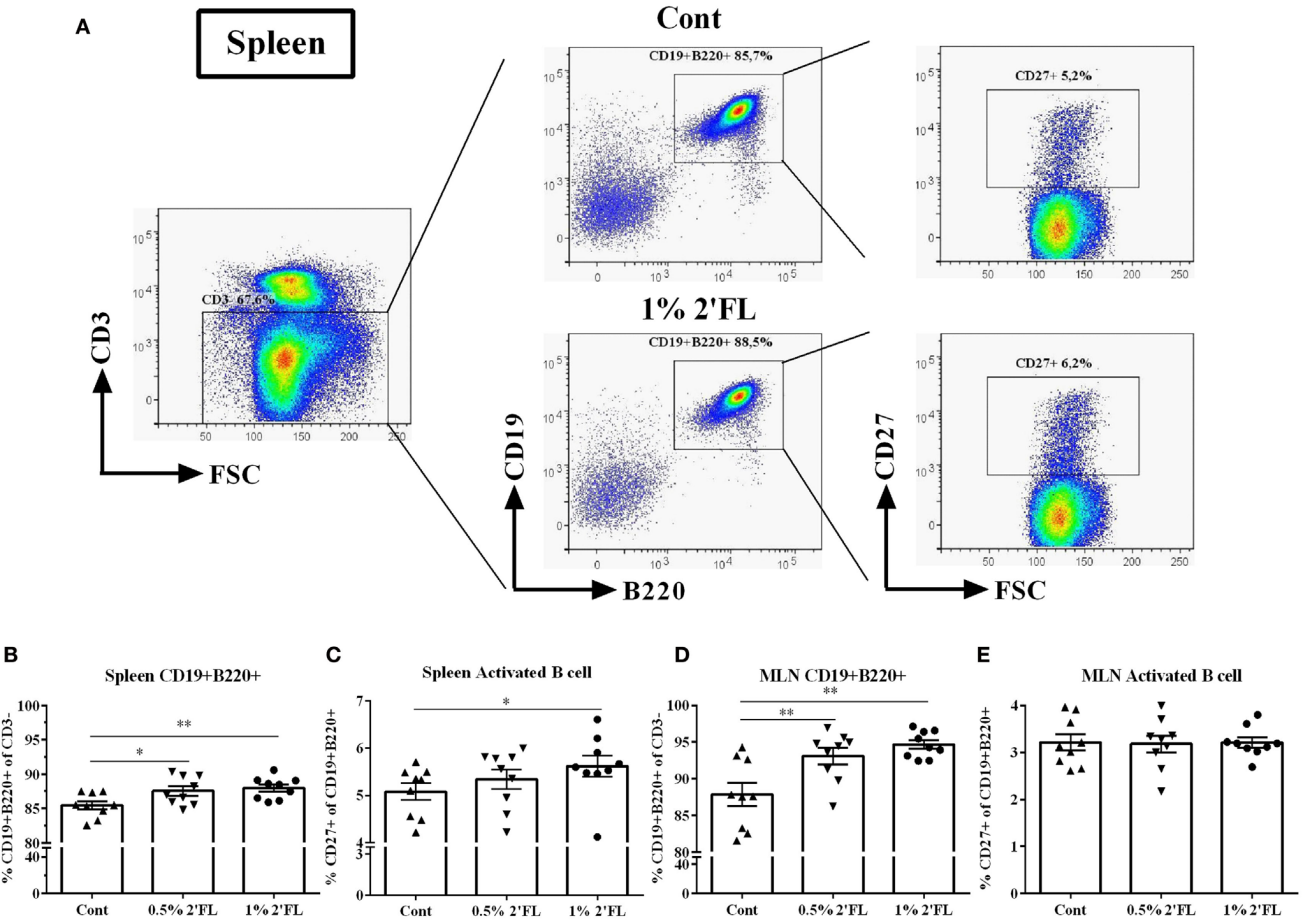
Flow cytometric analysis of B-cells subpopulations in spleen and mesenteric lymph node (MLN) collected at day 31. **(A)** Representative plots of B-cells in spleens from control (Cont) and 1% 2′-fucosyllactose (2′FL) groups. Percentage of CD19+ B220+ B-cells in the **(B)** spleen and **(D)** MLN. Percentage of memory B-cells (CD27+ of CD19+ B220+ cells) in the **(C)** spleen and **(E)** MLN. Data are presented as mean ± SEM (*n* = 9). *p < 0.05, **p < 0.01 are the significant differences as compared to control mice. One-way ANOVA followed by Bonferroni’s *post hoc* test was used for selected groups.

2′-Fucosyllactose mediated increase in antibody levels prompted us to investigate whether B-cell development was affected. To this end, the frequency and activation status of B-cells in isolated spleen and MLN cells from mice receiving 0.5 and 1% 2′FL were compared to vaccinated mice receiving control diet, using flow cytometry (Figure 2A, gating strategy). Surface marker expression analysis of CD19, CD220, and CD27 revealed a significantly higher frequency of CD19+ B220+ B-cells in spleens and MLNs of both the 0.5 and 1% 2′FL receiving mice compared to the control mice (Figure 2B, 2.1 and 2.5% increase by 0.5 and 1% 2′FL, *p* < 0.05, *p* < 0.01, respectively; Figure 2D, 6.8 and 5.2% increase by 0.5 and 1% 2′FL, *p* < 0.01, *p* < 0.01, respectively). CD27 expression has been used to distinguish between memory and naive B-cells (22). Although no effect on the percentage of CD19+ B220+ CD27+ activated B-cell in the MLN was detected (Figure 2E), there was a higher percentage (0.5%) of CD27+ activated CD19+ B220+ B-cells in spleen cells from vaccinated mice receiving 1% 2′FL compared to control vaccinated mice (Figure 2C, *p* < 0.05).

### Dietary 2′FL Differentially Activated DCs in the Spleen and MLN

**Figure 3:**
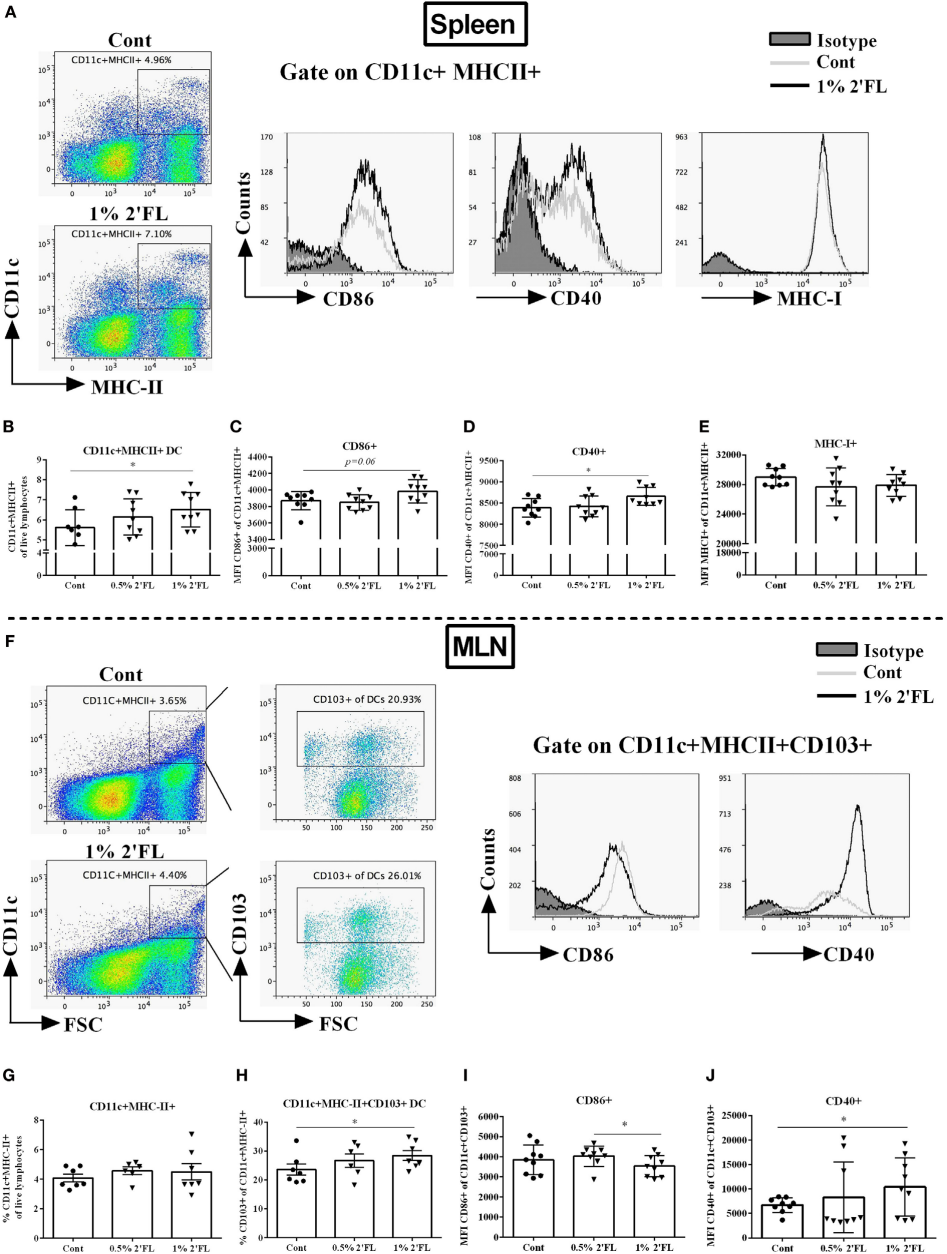
Flow cytometric analyses of dendritic cells (DCs) percentages and surface markers expression in the spleen and mesenteric lymph node (MLN) at day 31. Spleen DCs were analyzed based on their expression of CD11c and MHC-II. **(A)**. Representative plots and histogram of splenic DCs from control (Cont) and 1% 2′-fucosyllactose (2′FL) groups. Percentage of **(B)** CD11c+ MHCII+ DCs in total splenocytes, activation status was further distinguished based on their median fluorescence intensity (MFI) of surface markers **(C)** CD86, **(D)** CD40, and **(E)** MHC-I expression. **(F)** Representative plots and histogram of MLN-DC from control (Cont) and 1% 2′FL group. **(G)** Percentage of CD11c+ MHC-II+ in total MLN cells; **(H)** percentage of tolerogenic dendritic cells (tDCs) (CD103+ of CD11c+ MHC-II+ cells) in MLN-DC; MFI of surface markers **(I)** CD86 and **(J)** CD40 expression on tDCs. Data are presented as mean ± SEM of *n* = 6–9 mice per group; *p < 0.05, is depicted significant difference compared to control using one-way ANOVA followed by Kruskal–Wallis’ non-parametric test.

Knowing the central role of the DCs in presenting antigens to B-cells and activating the CD4+/8+ T-cell populations, we next studied the phenotype and activation status of DCs in the spleen and MLNs of mice receiving 2′FL. Fresh isolated splenocytes and MLN cells were stained with maturation (MHC-II, MHC-I) and costimulatory surface markers (CD40, CD86) and quantified using flow cytometry (Figure 3A). Around 1% increase of CD11c+ MHCII+ DCs was detected in splenocytes isolated from mice receiving 1% 2′FL compared to control mice (Figure 3B, *p* < 0.05). Moreover, the expressions of CD86+ (Figure 3C, *p* = 0.06) and CD40+ (Figure 3D, *p* < 0.05) on CD11c+ MHCII+ DCs as detected by median fluorescence intensity (MFI) were increased in mice receiving the 1% 2′FL diet. No differences were detected in the expression of MHC-I on the splenic DCs between 2′FL and control diet receiving mice (Figure 3E).

In the MLN, CD103+ DCs have been suggested to represent *bona fide* DCs due to their capacity to induce differentiation of naive CD4+ T-cells into Foxp3+ regulatory T-cells (Treg) (23) and driving responding T-cells to express the gut-homing molecules (24). Although the percentage of CD11c+ MHCII+ DCs in the MLN was not significantly different between dietary intervention groups (Figure 3F,G), a significant increase (5.4%) within the CD103+ population, the so-called tolerogenic DCs (tDCs, CD103+ CD11c+ MHC-II+ DCs) was found in mice receiving 1% dietary 2′FL vs. control mice (Figure 3H, *p* < 0.05). In addition, MFI of CD86+ expression on the CD103+ DCs was significantly decreased (Figure 3I, *p* < 0.05), whereas the MFI of CD40+ expression was significantly increased (Figure 3J, *p* < 0.05) on isolated MLNs from mice receiving 1% 2′FL dietary intervention compared to vaccinated control mice. These data indicate that 1% 2′FL supplementation in the diet induce more activated DCs in the spleen and a more tolerogenic phenotypic DCs in the MLN.

### Increased Th1 and Tregs Frequency in Spleen of Mice Receiving 2′FL

**Figure 4:**
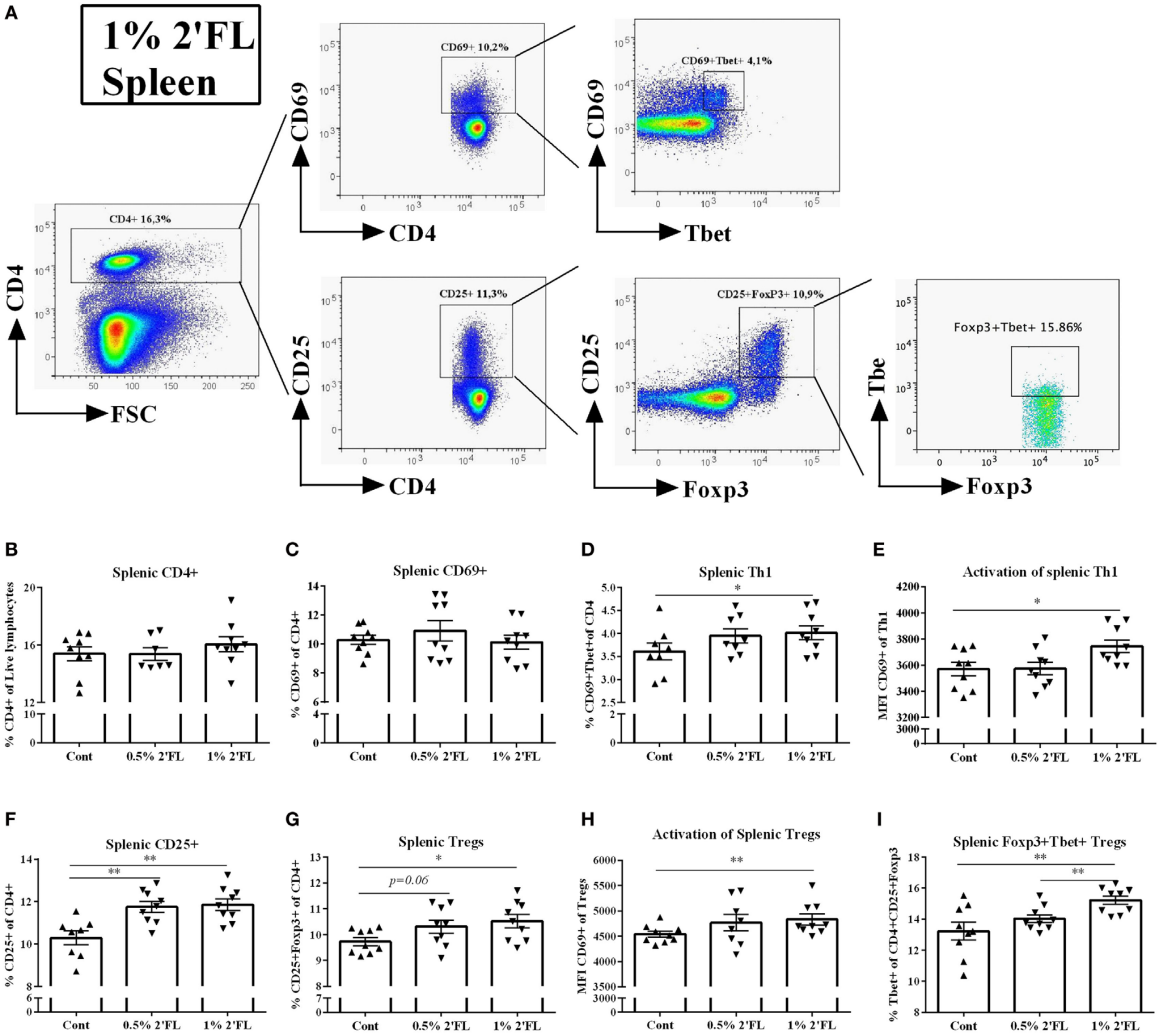
Flow cytometric analysis of T helpher (Th) 1 and regulatory T cell (Tregs) populations in the spleen at day 31. **(A)** Representative plots of splenic CD4 T-cells from 1% 2′FL group were shown. **(B)** Percentage of CD4+ T-cells in total live lymphocytes. Percentage of **(C)** early (CD69+ of CD4+ cells) and **(D)** late activated (CD25+ of CD4+ cells) CD4+ T-cells in the spleen **(F)**. Percentage of **(D)** Th1 cells (Tbet+ CD69+ of CD4+ cells) and **(G)** Tregs (Foxp3+ CD25+ of CD4+ cells) in CD4+ T-cell. Median fluorescence intensity (MFI) of CD69 expression of **(E)** Th1 and **(H)** Treg. **(I)** Percentage of Foxp3+ Tbet+ Tregs (Tbet+ of CD4+ CD25+ Foxp3+ cells). Data are presented as mean ± SEM of *n* = 7–9/group; *p < 0.05, **p < 0.01 are significant differences compared to control using Kruskal–Wallis’ non-parametric test, followed by Dunn’s *post hoc* test for selected pairs.

Changes in DC phenotype may directly influence antigen presenting capacity and subsequently the specific T-cell responses. To test whether T-cell specific vaccine responses were modulated by 2′FL in a Th1-skewed fashion [important to improve the vaccine efficacy (25)], freshly isolated splenocytes and MLNs were stained with intracellular transcription factor Tbet (Th1) and Foxp3 (Treg) (Figure 4A). In the spleen, no differences in frequency of either CD4+ or CD4+ CD69+ T-cells were detected (Figures 4B,C). However, Tbet+ of CD4+ CD69+ T-cell (activated Th1 type of cells) was increased by 1% 2′FL compared to vaccinated mice receiving control diet (Figure 4D, *p* < 0.05). In addition, the Tbet+ expression on CD4+ CD69+ cells was upregulated (*p* < 0.01, data not shown), and the Tbet+ CD69+ CD4+ T-cell activation, as measured by the MFI of CD69 expression was increased in mice receiving 1% dietary 2′FL compared to vaccinated mice receiving control diet (*p* < 0.05, Figure 4E).

Conversely, both dosages of 2′FL slightly increased the percentage of splenic CD25+ CD4+ T-cells (1.4 and 1.6% increase by 0.5 and 1% 2′FL, respectively, Figure 4F, *p* < 0.01 for 0.5% 2′FL, and *p* < 0.001 for 1% 2′FL) as well as the percentage (0.6 and 0.8% increase by 0.5 and 1% 2′FL, respectively, Figure 4G, *p* = 0.06 for 0.5% 2′FL, *p* < 0.01 for 1% 2′FL) and activation marker expression CD69 (*p* < 0.01 for 1% 2′FL, Figure 4H) of Foxp3+ Tregs as compared to control diet. Furthermore, a 2% increase of Foxp3+ Tbet+ Tregs in the spleen of 1% 2′FL mice was detected (Figure 4I, *p* < 0.05). Similar 2′FL effects on the T-cell subpopulations in the MLN are shown in Figure S1 in Supplementary Material.

### Vaccine-Specific CD4+ and CD8+ T-Cell Proliferation Increased in Mice Receiving Dietary 2′FL upon *Ex Vivo* Restimulation

**Figure 5:**
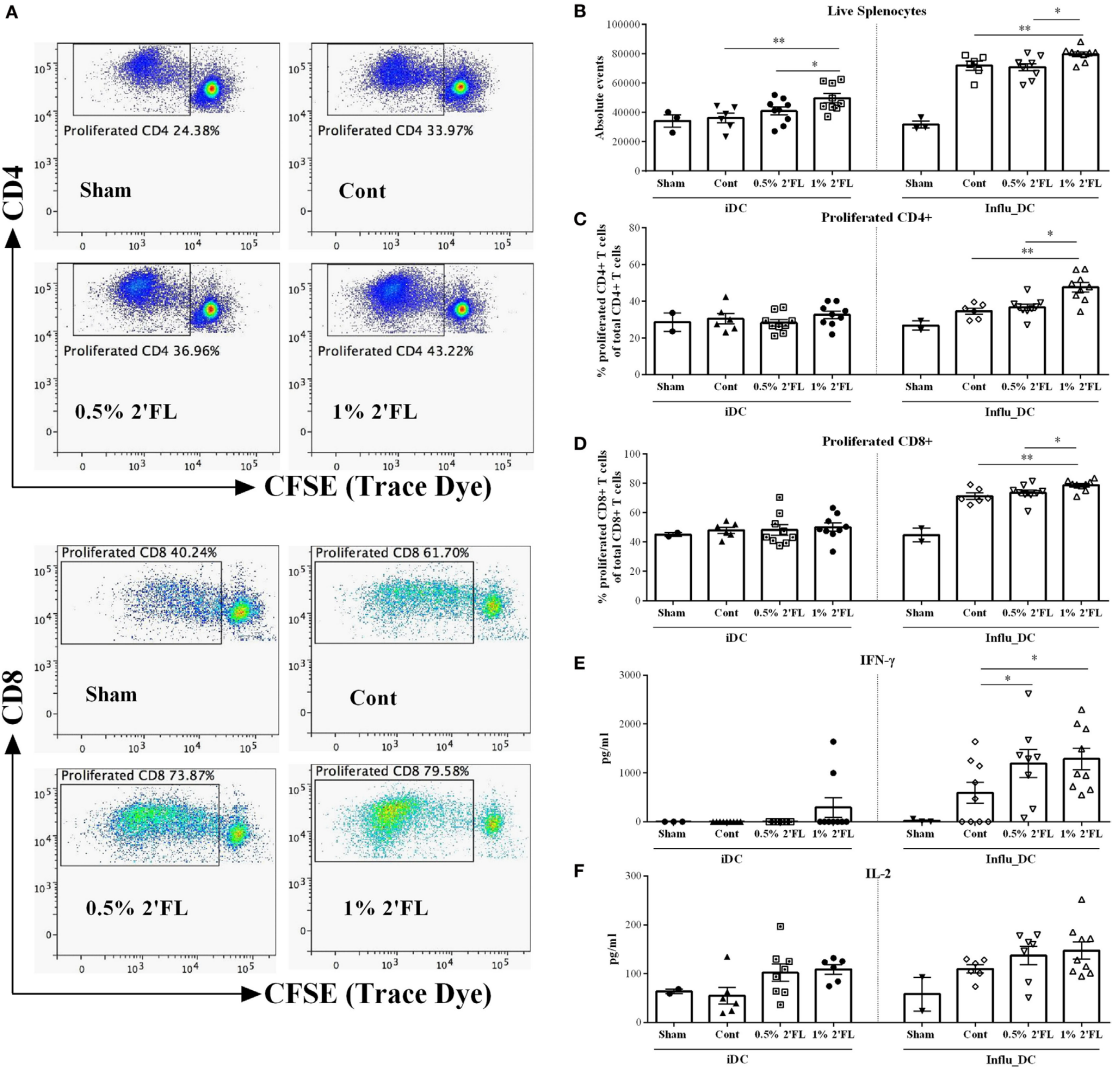
Vaccine-specific proliferation and cytokines production after *ex vivo* restimulation with influenza-loaded bone marrow-derived dendritic cells (BMDCs). **(A)** Representative plots of proliferated CD4+ and CD8+ T-cells after *ex vivo* restimulation. **(B)** Absolute events of live splenocytes after *ex vivo* restimulation. Percentage of proliferated **(C)** CD4+ and **(D)** CD8+ T-cells after *ex vivo* restimulation. Production of **(E)** IFN-γ and **(F)** IL-2 after *ex vivo* restimulation. BMDCs were derived prior to the section at day 25, and loaded with or without 0.9 μg/mL influvac for 24 h, at day 31, BMDCs were cocultured with CFSE-labeled whole spleen cells from sham or vaccinated mice at a ratio of 1:10 for 5 days, fresh spleen suspensions were derived from spleen samples collected at day 31. Cocultured cells were stained with viability dye (APC-cy7), CD4 (APC), and CD8 (PE) antibody before FACS measurement, cell proliferation was monitored by levels of CFSE dilution. In a parallel experiment, influenza-loaded BMDCs were cocultured with whole spleen cells from sham or vaccinated mice at a ratio of 1:10 for 5 days, supernatant was collected at day 5. Interferon-γ (IFN-γ) was measured by ELISA; IL-2 was measured by Biolegend assay. Data are presented as mean ± SEM of *n* = 2–3/group in sham group, and *n* = 6–9/group in other groups; Sham group (*n* = 3) was never used for statistical comparisons to supplemented groups, but served solely to demonstrate the specificity of vaccine-induced responses. *p < 0.05, **p < 0.01 are significantly different as compared to control using Kruskal–Wallis’ non-parametric test, followed by Dunn’s *post hoc* test for selected pairs.

To investigate whether the altered T-cell responses in the spleen are vaccine-specific, *ex vivo* restimulation was performed. CFSE-labeled whole spleen cells from sham or vaccinated mice were cocultured with BMDCs loaded with or without influenza (the same batch used in the primary and booster vaccinations), total live lymphocytes, proliferation of CD4+ and CD8+ T-cells were assessed by flow cytometry after 5 days of coculture (gating strategy Figure 5A). Surprisingly, by coculturing with control DCs, the absolute number of overall splenocytes was increased by vaccinate mice receiving 1% 2′FL compared to vaccinated control mice (Figure 5B, *p* < 0.01) and 0.5% 2′FL (Figure 5B, *p* < 0.05), whereas no effects on the percentage of proliferation of CD4+ and CD8+ T-cells by dietary intervention were observed when cocultured with control DCs (Figures 5C,D). Upon restimulation by influenza-loaded DCs, low background responses were shown for all three parameters (namely, number of total splenocytes, percentage of CD4+ and CD8+ T-cell) in the sham-vaccinated mice. All vaccinated groups showed significantly higher number of total live cells compared to sham mice, indicating that the responses were vaccine-specific. Vaccinated-mice receiving 1% 2′FL had even higher absolute number of live cells compared to control vaccinated mice (Figure 5B, *p* < 0.01). 13% higher CD4+ and 7.4% higher CD8+ T-cells in the 1% 2′FL receiving group were detected in comparison to control vaccinated group (Figure 5C, *p* < 0.01; Figure 5D, *p* < 0.001, respectively), whereas changes within the 0.5% 2′FL did not reach significance. These data indicate that dietary intervention with 2′FL next to B-cell development also increased vaccine-specific T-cell responses.

In parallel with these proliferation assays, unlabeled splenocytes were cocultured with BMDCs loaded with or without influenza and vaccine-induced cytokine production were determined in the cell supernatant collected at day 5. In line with observed T-cell changes in the spleen, both 0.5 and 1% 2′FL diets induced higher IFN-γ production of the spleen cells compared to control diet (Figure 5E, *p* < 0.05, *p* < 0.05, respectively), and a tendency toward increased levels of IL-2 after vaccine restimulation was observed (Figure 5F).

### Dietary 2′FL Differentially Changed Intestinal mRNA Expression

**Figure 6:**
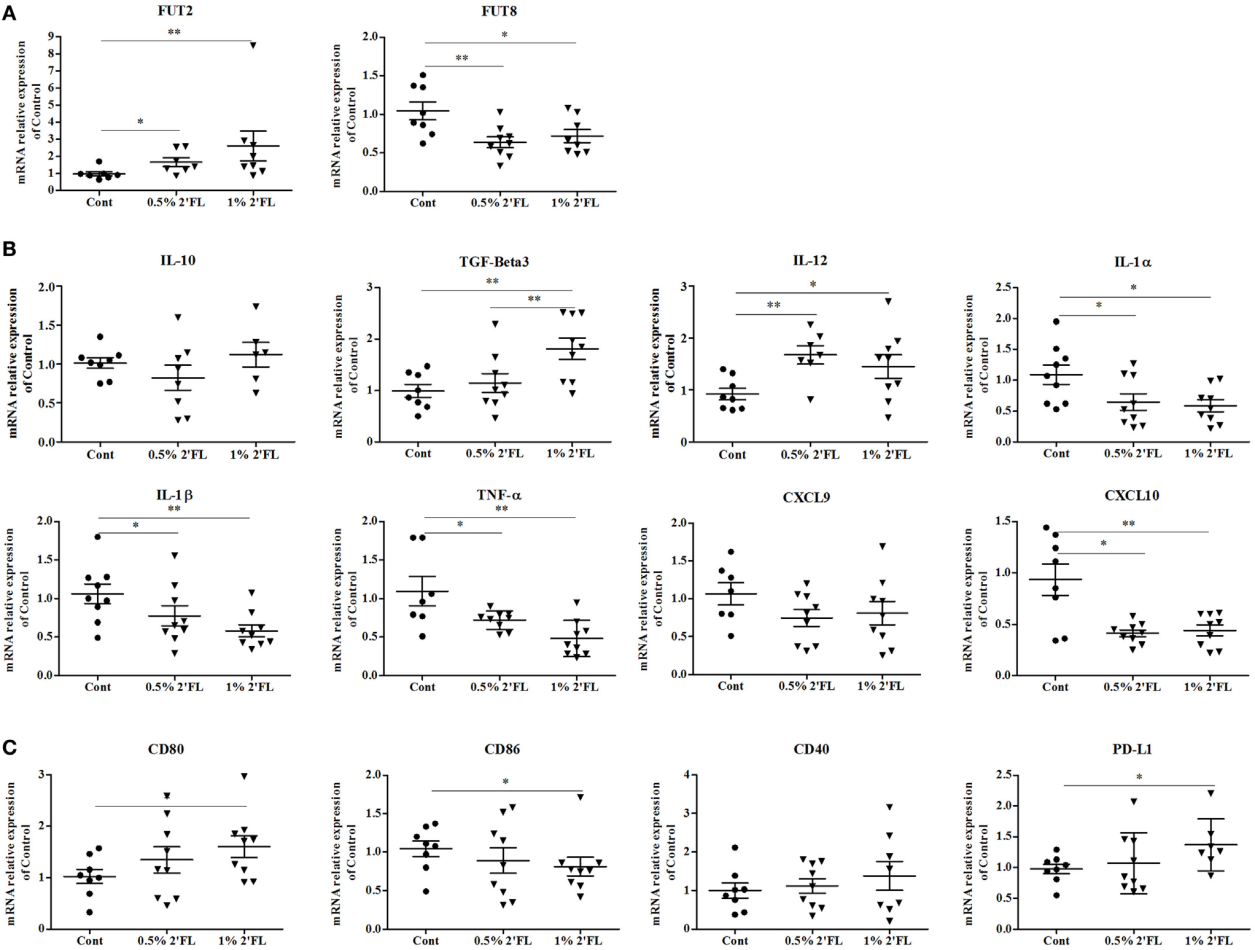
Impact of the 2′-fucosyllactose (2′FL) dietary intervention on mRNA expression in ileum collected at day 31. Relative expression of **(A)** fucosyltransferases-related genes including fucosyltransferase (FUT) 2 and FUT8, **(B)** anti- (TGF-beta3, IL-10) and proinflammatory cytokines (IL-12β, IL-1α, IL-1β, TNF-α) and chemokines (CXCL10, CXCL9)-related gene expression, and **(C)** Costimulatory (CD80, CD86, CD40) and inhibitory (PD-L1) markers gene. Data are presented as mean ± SEM of *n* = 6–9/group; *p < 0.05, **p < 0.01, ***p < 0.001 indicate significant differences. Kruskal–Wallis’ non-parametric test, followed by Dunn’s *post hoc* test for selected pairs was used.

To detect effects of dietary 2′FL on the intestinal inflammation, ileal mRNA expression profiles were analyzed. 2′FL is a FUT2-dependent HMOS and FUT2 is a gene that encodes α (1,2)-fucosyltransferase which has been implicated to decreased risk of infectious diseases (26). Here, we detected a significant increase in mRNA expression of FUT2 in the ileum of mice receiving 2′FL (Figure 6A, *p* < 0.05 for 0.5% 2′FL, *p* < 0.01 for 1% 2′FL). On the other hand, mRNA levels of FUT8 was significantly down-regulated in mice receiving 2′FL (Figure 6A, *p* < 0.001 for 0.5% 2′FL, *p* < 0.05 for 1% 2′FL).

In order to detect 2′FL specific anti-inflammatory properties on human intestinal cells (27), both anti- and proinflammatory cytokine-related genes were measured. As shown in Figure 6B, no effect of the dietary interventions was distinguished on IL-10 mRNA expression, however, an increase in the mRNA expression of TGF-β3 was detected (*p* < 0.01 for 1% 2′FL). mRNA expression of (pro-)inflammatory cytokine-related genes, namely IL-1α, IL-1β, TNF-α, and CXCL-10, except for CXCL-9, were significantly decreased by dietary 2′FL. Interestingly, mRNA expression of IL-12β was also enhanced, which seems associated with the DCs activation status shown in Figure 6C. Moreover, the qPCR data revealed reduced CD86 and increased CD80 mRNA levels in the ileum by dietary 2′FL (Figure 6C). The increased PD-L1 mRNA confirms the tolerogenic phenotype of intestinal DCs since the expression of PD-L1 has been correlated with induction of Foxp3+ Tregs (28).

### 2′FL Directly Modulate the Phenotype and Antigen-Presenting Capacity of BMDCs *In Vitro*

**Figure 7:**
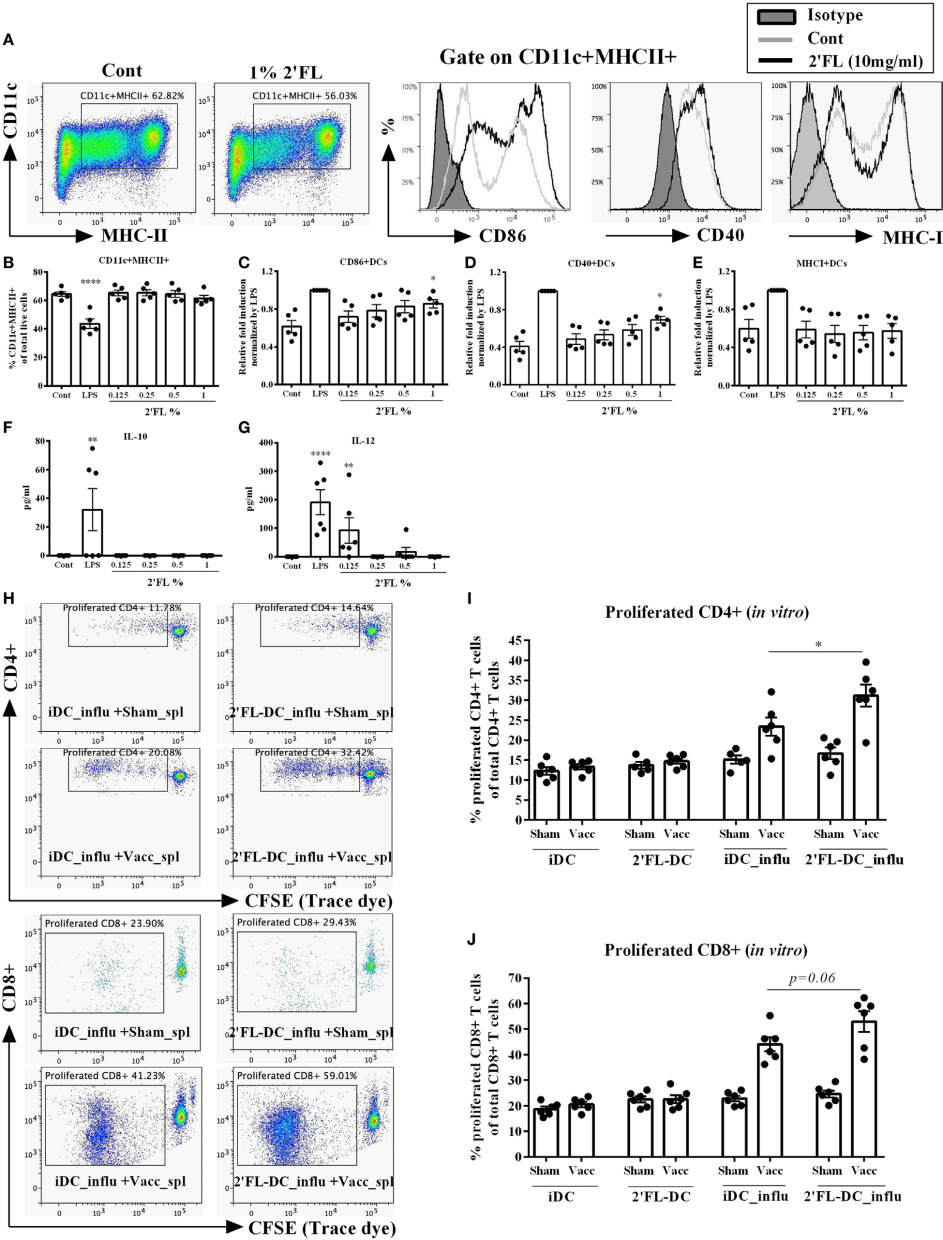
Effects of 2′-fucosyllactose (2′FL) on the phenotypes and antigen presenting capacity of bone marrow-derived dendritic cell (BMDC) *in vitro*. **(A)**. Representative plots and histogram of splenic dendritic cells (DCs) from untreated control (Cont) and 1% 2′FL, LPS (100 ng/mL) was used as a positive control. Percentage of **(B)** CD11c+ MHCII+ DCs in total live cells, maturation status was further distinguished based on their percentage of surface markers **(C)** CD86, **(D)** CD40, and **(E)** MHC-I positive expression. **(F)** IL-10 and **(G)** IL-12 production by BMDCs. Immature BMDCs were generated and treated with 0.125, 0.25, 0.5, or 1% 2′FL for 24 h, medium and LPS stimulation was used as negative and positive control, respectively. Cells were collected for analyzing the phenotypes of BMDCs by flow cytometry; supernatant was collected for the measurement of IL-10 and IL-12p70 by ELISA. **(H)** Representative plots of proliferated CD4+ and CD8+ T-cells after *ex vivo* restimulation by different DCs. Percentage of proliferated **(I)** CD4+ and **(J)** CD8+ T-cells after *ex vivo* restimulation by different DCs. Immature BMDCs were treated with either medium or 1% 2′FL for 5 h before being loaded with 0.9 μg/mL influvac for 24 h, afterward different BMDCs were cocultured with CFSE-labeled fresh whole spleen cells from non-vaccinated (sham) or vaccinated mice at a ratio of 1:10 for 5 days, fresh spleen suspensions were derived from spleen samples of vaccinated donor mice at day 31. Cocultured cells were stained with CD4 (APC) and CD8 (PE) antibody before FACS measurement, cell proliferation was monitored by levels of CFSE dilution. Data are presented as mean ± SEM, six independent experiments were performed; *p < 0.05, **p < 0.01, ***p < 0.001, ****p < 0.0001. Kruskal–Wallis’ non-parametric test was used for panels **(B–G)** Kruskal–Wallis’ non-parametric test, followed by Dunn’s *post hoc* test for selected pairs was used for panels **(I,J)**. iDC-influ = immature DCs loaded with influvac; 2′FL-DC_influ = 1% 2′FL treated DC loaded with influvac; Sham_spl = spleen cells from sham mice; Vacc_spl = spleen cells from vaccinated mice.

Since HMOS are also potential microbiota modulators, it is still unclear whether the observed *in vivo* immunomodulatory effects of dietary 2′FL are direct and/or microbiota-dependent. To study the direct effect of 2′FL on immune regulation, an *in vitro* study in which BMDCs were treated by four different concentrations [0.125, 0.25, 0.5, and 1% (w/v in medium)] of 2′FL was performed. Medium un-treated and LPS (100 ng/mL) treated cells were used as negative and positive control, respectively. Using the same analysis strategy as used for the *in vivo* experiment, DCs were first distinguished by gating at CD11c+ MHCII+ cells (Figure 7A). 2′FL had no effects on percentage of CD11c+ MHCII+ DCs compared to untreated control cells (Figure 7B). The percentage of CD40+ and CD86+ expression on BMDCs was increased (0.29 relative fold and 0.24 relative fold, respectively) by the 1% 2′FL (Figures 7C,D), whereas only the MFI of CD86+ expression was significantly elevated by 1% 2′FL (Figure S2A in Supplementary Material, 0.26 relative fold increase, *p* < 0.01). Although the percentage of MHC-I was not changed (Figure 7E), the MFI of MHC-I expression on BMDCs was significantly increased by 1% 2′FL (Figure S2C in Supplementary Material, 0.47 relative fold increase, *p* < 0.05). 2′FL had no effects on the IL-10 production (Figure 7F) but increased IL-12p70 production at the lowest concentration (Figure 7G, *p* < 0.01) compared to untreated control were detected.

To determine the antigen presenting capacity of 2′FL modulated BMDCs, *in vitro* restimulation assay was performed. BMDCs were treated with 1% 2′FL since this concentration induced maximal phenotypical changes as shown in Figures 7C–E, thereafter BMDCs were loaded with or without 0.9 µg/mL influvac for 24 h before being cocultured with fresh whole splenocytes from non-vaccinated (sham) or influenza-vaccinated mice. Proliferation of CD4+ and CD8+ T cells were measured by flow cytometry (Figure 7H). 1% 2′FL treated BMDCs induced 7.7% higher proliferation of vaccine-specific CD4+ T-cells compared to medium untreated BMDCs (Figure 7I, *p* < 0.05). Besides, 1% 2′FL treated BMDCs induced an increasing tendency of proliferated CD8+ T-cells (Figure 7J, *p* = 0.06). These findings are consistent with *in vivo* data, supporting direct effects of 2′FL on phenotype and function of antigen presenting DCs and consequently improved immune development, resulting in enhanced vaccine response.

## Discussion

Within this study, we demonstrate that dietary 2′FL, dose-dependently improve both cellular and humoral influenza vaccine responses in a murine vaccination model. Followed by *in vitro* confirmation of a microbiota-independent direct immunomodulatory property of 2′FL on antigen presenting DCs. Specific HMOS structures have been shown to be linked to a reduction in the risk of food allergy (29, 30) and infectious episodes (31). The immunological consequences of adding this single dietary component normally present within a complex matrix of HMOS were studied in an established immune system. To our knowledge, this is the first demonstration of a specific HMO capable to directly improve vaccine responsiveness in an influenza vaccination murine model.

Efficacy of neonatal vaccination is expressed in correlation of protection, and/or vaccine specific antibody levels (25). Limitations in establishment of proper early-life IgG antibody responses in terms of both quantity and quality may be due to intrinsic limitation of B-cells in development (32). Therefore inducing improved B-cell responses is seen as a potential strategy toward improved vaccine efficacy. Here, we identify dietary 2′FL to be capable to enhance both the B-cell frequency and activation (Figure 2), resulting in improved vaccine specific humoral responses as observed by enhanced levels of IgG1 and IgG2a in mice (Figures 1D,E). However, unlike the present study, previous studies in which prebiotic scGOS/lcFOS improved the influenza vaccine-specific DTH responses, limited to no effects on vaccine-specific IgG1 and IgG2a antibody responses were detected (17), indicating a different immunomodulatory mechanism by which dietary 2′FL now seems to improve the vaccine efficacy in mice. This vaccination model is well suited to establish the capacity of one’s immune system to respond to a given antigen, which is a very good way to study immune modulation by nutrition (33). In order to determine potential use and translational capacity of our findings, further research is warranted. In human studies, decreased numbers of memory B-cells (CD27+ IgA+, CD27+ IgM+ and CD27− IgG+ memory B-cells) were detected in blood samples of healthy breastfed children without notification of secretor status (34). On the other hand, the presence of activated B-cells that are primed to secret IgG (35), as well as specific antibodies against viral or bacterial antigens (36) are present in breast milk. Similar to our study, other components present in human milk, have been found to influence vaccination responses directly. For instance, a diet enriched in (n-3) lcPUFA contributes to a healthy immune response using same *in vivo* models (37) as well as models with altered B-cell activity (38). Bioactive components such as 2′FL present within the complex mixture of HMOS, can stimulate B-cell development and subsequent antibody production, which are known human milk induced immune benefits. We therefore suggest, that additional research is needed to see what the contribution of 2′FL alone is in combination with other prebiotic oligosaccharides, and nutritional components. In addition, future clinical studies are warranted to further prove effect of dietary 2′FL in the developing immune system of infants, immune compromised population and/or the elderly.

Dendritic cells are professional antigen-presenting cells that initiate and prime adaptive immune response, and thus are critically important in the induction of vaccine-specific cellular immune responses (14). The DCs maturation status, which is characterized by an increase in cell surface expression of MHC-I and MHC-II molecules and costimulatory molecules including CD40, CD86, increased antigen presenting capacity and induction of specific cytokine production (39), is of great importance to lead to an optimized ability to initiate T-cell immunity. In the current study, dietary 2′FL induced a higher percentage of mature CD11+ MHC-II+ DCs in the spleen of the vaccinated mice (Figures 3B–D). However, since metabolites from bacterial HMOS degradation, such as SCFAs can directly interact with both human and murine DCs (40), it was not clear whether the maturation effects of dietary 2′FL on the DCs were directly mediated or derived through a microbiota-dependent mechanism. Our *in vitro* data show an 2′FL concentration-dependently increase in the expression of CD40 and CD86 on the CD11c+ MHCII+ DCs (Figures 7C–E), and antigen-presenting function with increased proliferation capacity of antigen-specific CD4+ and CD8+ T-cells (Figures 7I,J). These findings together confirm that 2′FL directly interacts with antigen presenting DCs. This notion is supported by the evidence that 2′FL binds to DC-SIGN, which is expressed on DCs (15). However, whether or not 2′FL interacts with DC-SIGN, as well as if the DC-SIGN-mediated signaling is responsible for the observed immune regulation needs to be further investigated.

The functional changes of DCs were subsequently reflected by the induction of higher percentage of Tbet-expressing CD4+ T-cells in the spleen of mice receiving dietary 2′FL (Figure 4D). This is in line with previous studies where dietary supplementation of prebiotic oligosaccharides enhanced systemic Th1-skewed cytokines in a murine vaccination mouse model (19). In the same vaccination model, scGOS/lcFOS supplementation increases systemic Th1 responses as seen by increased percentage of Tbet+ activated CD69+ CD4+ T-cell (18). Our findings together with these studies, underscore the critical role of immune-modulating and, more specifically, Th1-skewed effect of dietary oligosaccharides in the improvement of vaccine responsiveness. Furthermore, *ex vivo* restimulation of splenocytes from 2′FL-fed vaccinated mice by influenza-loaded BMDCs results in a higher proliferation of vaccine-specific CD4+ and CD8+ T-cell compared to control vaccinated mice (Figures 5C,D), accompanied by a higher production of IFN-γ (Figure 5E). These results indicate that dietary 2′FL equipped the vaccinated mice with the capacity to effectively and sustainably expand vaccine-specific CD4+ and/or CD8+ T-cells producing IFN-γ upon encountering corresponding antigens. Thus, next to the innate immunity, dietary 2′FL also enhances the adaptive immunity in this murine vaccination model, indicating the potential of 2′FL in the acquisition of immune competence during early life of infants.

Nutritional factors are regulators of gene expression in mammals, it is therefore reasonable to assume that one of the possible explanations for the vaccination response improving effects of the dietary 2′FL is modulation of gene expression. Here, we reported that dietary 2′FL up-regulated intestinal FUT2 gene expression while down-regulated FUT8 gene expression (Figure 6A), both of which encode fucosyltransferase enzymes, indicating the impact of dietary 2′FL on the metabolism of the vaccinated mice. The adapted metabolism may subsequently lead to initiating an intracellular signaling pathway that coordinately regulates this set of genes. Our observation that anti- or proinflammatory cytokines production-related genes, as well as costimulatory signaling-related genes were changed seem to reflect the metabolic changes. In the human intestine, FUT2 gene expression has been strongly associated with the diversity and composition of Bifidobacterial population (41), which has been associated with improvement of the vaccine-specific DTH responses by scGOS/lcFOS supplementation (19). However, the direct relation between alteration of these gene expressions with improved vaccination efficacy needs to be further investigated.

Human milk is a well-established factor to strengthen the defense against infections in infants (42–44), and 2′FL is a principal component of the complex HMOS mixture present in human milk. In the current study, we demonstrated an improvement of vaccine-specific DTH and antibody responses by dietary 2′FL. The stimulatory effects were associated with activation of memory B-cells, induction of optimal Th1 responses *via* maturing DCs in the spleen, and facilitation of expanding vaccine-specific splenic CD4+ and CD8+ T-cells upon restimulation *ex vivo*. These data together with the *in vitro* data indicate the presence of a microbiota-independent direct immune modulatory potential of 2′FL, and thus support the notification of health beneficial properties derived from complex mixture of oligosaccharides within human milk.

## Ethics Statement

Experimental procedures were approved by the Ethical Committee of Animal Research of Utrecht University and the Central Commission for Animal use (approval numbers: DEC2015.II.243.038 and AVD108002016460) and complied with the principles of good laboratory animal care following the European Directive for the protection of animals used for scientific purposes.

## Author Contributions

LX, BL, and GF designed the experiments; LX, TLM, IA, NAH, NK, BB, and SO performed experimental procedures; LX performed data collection, analysis and drafted the manuscript; JG, GF, and BL supervised the program; BS made specific contributions to the program with regard to the Human milk oligosaccharides. All authors listed have made significant contribution to writing the manuscript and approved it for publication.

## Conflict of Interest Statement

None of the authors have a competing financial interest in relation to the presented work; JG is head of the Division of Pharmacology, Utrecht Institute for Pharmaceutical Sciences, Faculty of Science at the Utrecht University, and partly employed by Nutricia Research. Both BS and BL are employed by Nutricia Research. BL, as indicated by the affiliations, is leading a strategic alliance between University Medical Centre Utrecht/Wilhelmina Children’s Hospital and Nutricia Research.

## Supplementary Material

Figure S1 Flow cytometric analysis of T helper (Th) 1 and regulatory T cells (Tregs) populations in the mesenteric lymph node (MLN). **(A)** Percentage of CD4+ T cells of total live lymphocytes. Percentage of **(B)** early (CD4+ CD69+ cells) and **(E)** late activated (CD4+ CD25+ cells) CD4+ T cells in the MLN. Percentage of **(C)** Th1 cells (Tbet+ of CD4+ CD69+ cells) and **(F)** Tregs (Foxp3+ of CD4+ CD25+ cells) in the MLN. Activation of Th1 **(D)** and Treg **(G)** was presented by the MFI of CD69 expression. **(H)** Percentage of Foxp3+ Tbet+ Tregs (Tbet+ of CD4+ CD25+ Foxp3+ cells). Briefly, No differences between the dietary intervention groups on the percentage of CD4+ and CD4+ CD69+ T cells **(B)** in the MLN were studied. Significantly lower percentages of Th1 cells (Tbet+ CD69+ CD4+ T-cell) were observed in both 0.5 and 1% 2′-fucosyllactose (2′FL) supplemented groups compared to control group, although the activation status as shown by the MFI of CD69+ expression of cells did not differ between groups. In line with the observed increased frequency of CD103+ DCs in the MLN, both 0.5 and 1% 2′FL groups displayed increased percentages of CD4+ CD25+ T-cell and Foxp3+ Tregs compared to control group. The activation status as shown by the MFI of CD69+ expression of Tregs and the percentage of Foxp3+ Tbet+ Tregs remained unaffected by dietary 2′FL in the MLN. Data are presented as mean ± SEM of *n* = 7–9/group; **p* < 0.05, ***p* < 0.01, ****p* < 0.001, and *****p* < 0.0001 one-way ANOVA followed by Bonferroni’s *post hoc* test for selected groups.

Figure S2 Median fluorescence intensity (MFI) of surface markers **(A)** CD86, **(B)** CD40, and **(C)** MHC-I expression on bone marrow-derived dendritic cells (BMDCs) treated by medium, LPS (0.125–1%) 2′-fucosyllactose (2′FL). The *y*-axis of the column bar graphs shows the relative expression of surface markers obtained by setting medium control as onefold within one experiment and for each donor. Data are presented as mean ± SEM, four independent experiments were performed; **p* < 0.05, ***p* < 0.01, and ****p* < 0.001. Kruskal–Wallis’ non-parametric test was used.

## Abbreviations

2′FL, 2′-fucosyllactose; BMDCs, bone marrow-derived dendritic cells; DCs, dendritic cells; DC-SIGN, DC-specific intercellular adhesion molecule-3-grabbing non-integrin; DTH, delayed-type hypersensitivity; lcFOS, long-chain fructo-oligosaccharides; Foxp3, forkhead box protein 3; FUT2, fucosyltransferase 2; FUT8, fucosyltransferase 8; HMOS, human milk oligosaccharides; i.d., intradermal; IFNγ, interferon-γ; Ig, immunoglobulin; MFI, median fluorescence intensity; MLN, mesenteric lymph node; SCFAs, short chain fatty acids; scGOS, short-chain galacto-oligosaccharides; s.c., subcutaneous; Th, T-helper; Treg, regulatory T cell.
