# Supplementary figures and images for: Human Milk Oligosaccharide 2′-Fucosyllactose Improves Innate and Adaptive Immunity in an Influenza-Specific Murine Vaccination Model
Source: Front Immunol. 2018 Mar 9;9:452. doi: 10.3389/fimmu.2018.00452 (PMC5854647; doi:10.3389/fimmu.2018.00452)

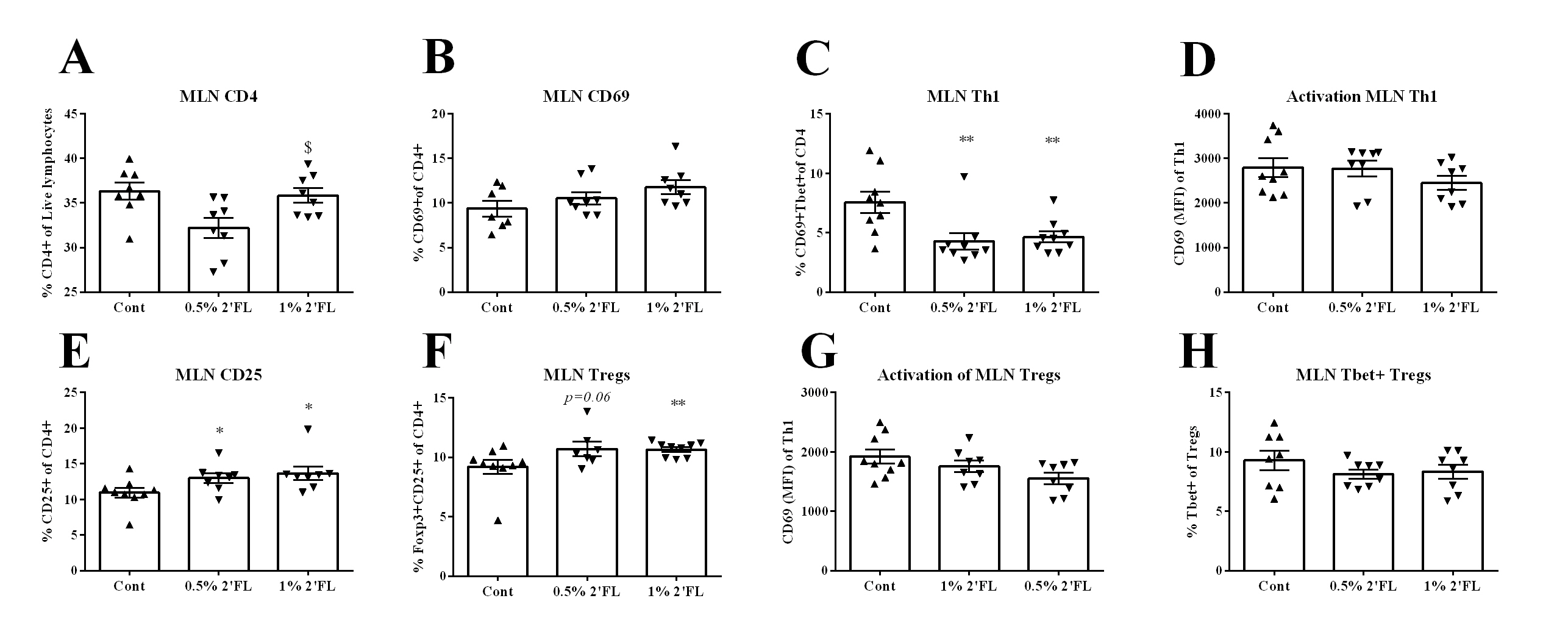

Supplement: Figure S1 — Flow cytometric analysis of T helper (Th) 1 and regulatory T cells (Tregs) populations in the mesenteric lymph node (MLN). (A) Percentage of CD4+ T cells of total live lymphocytes. Percentage of (B) early (CD4+ CD69+ cells) and (E) late activated (CD4+ CD25+ cells) CD4+ T cells in the MLN. Percentage of (C) Th1 cells (Tbet+ of CD4+ CD69+ cells) and (F) Tregs (Foxp3+ of CD4+ CD25+ cells) in the MLN. Activation of Th1 (D) and Treg (G) was presented by the MFI of CD69 expression. (H) Percentage of Foxp3+ Tbet+ Tregs (Tbet+ of CD4+ CD25+ Foxp3+ cells). Briefly, No differences between the dietary intervention groups on the percentage of CD4+ and CD4+ CD69+ T cells (B) in the MLN were studied. Significantly lower percentages of Th1 cells (Tbet+ CD69+ CD4+ T-cell) were observed in both 0.5 and 1% 2′-fucosyllactose (2′FL) supplemented groups compared to control group, although the activation status as shown by the MFI of CD69+ expression of cells did not differ between groups. In line with the observed increased frequency of CD103+ DCs in the MLN, both 0.5 and 1% 2′FL groups displayed increased percentages of CD4+ CD25+ T-cell and Foxp3+ Tregs compared to control group. The activation status as shown by the MFI of CD69+ expression of Tregs and the percentage of Foxp3+ Tbet+ Tregs remained unaffected by dietary 2′FL in the MLN. Data are presented as mean ± SEM of n = 7–9/group; *p < 0.05, **p < 0.01, ***p < 0.001, and ****p < 0.0001 one-way ANOVA followed by Bonferroni’s post hoc test for selected groups. [file image_1.jpeg]

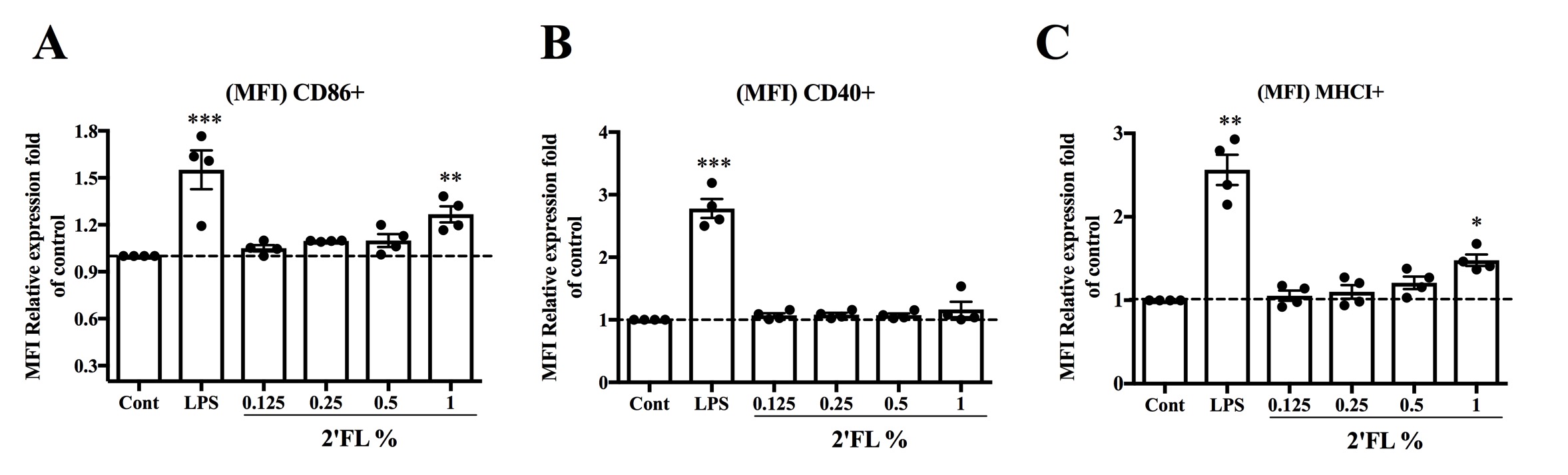

Supplement: Figure S2 — Median fluorescence intensity (MFI) of surface markers (A) CD86, (B) CD40, and (C) MHC-I expression on bone marrow-derived dendritic cells (BMDCs) treated by medium, LPS (0.125–1%) 2′-fucosyllactose (2′FL). The y-axis of the column bar graphs shows the relative expression of surface markers obtained by setting medium control as onefold within one experiment and for each donor. Data are presented as mean ± SEM, four independent experiments were performed; *p < 0.05, **p < 0.01, and ***p < 0.001. Kruskal–Wallis’ non-parametric test was used. [file image_2.jpeg]
